# Effect of the *Sargassum angustifolium* Extract on Methamphetamine-Induced Cytotoxicity in SH-SY5Y Cells

**DOI:** 10.1155/2022/9978235

**Published:** 2022-09-17

**Authors:** Zahra Salari, Mehdi Abbasnejad, Majid Askari Hesni, Saeed Esmaeili-Mahani

**Affiliations:** ^1^Department of Natural Resources, Baft Branch, Islamic Azad University, Baft, Iran; ^2^Department of Biology, Faculty of Science, Shahid Bahonar University of Kerman, Kerman, Iran

## Abstract

This study aimed to assess the effect of the *Sargassum angustifolium* extract in methamphetamine-induced SH-SY5Y cells death. The brown algae S. *angustifolium* was extracted with 80% ethanol. The SH-SY5Y cells were treated with different concentrations of methamphetamine to measure IC_50_.. The MTT test was used to assess the toxic effect of the *S. angustifolium* extract in SH-SY5Y cells. SH-SY5Y cells' survival was measured while cells were treated with different concentrations of methamphetamine and S. *angustifolium* extract simultaneously. A specific kit measured intracellular ROS levels. Western blot analysis evaluated the expression of cytochrome C and Bax/Bcl2 ratio. The results showed that 5 mM methamphetamine approximately killed 50% of the cells, so it is considered IC_50_. The MTT test showed no toxicity effect for the S. *angustifolium* extract. 80, 160, 320, and 640 *μ*g/ml of S. *angustifolium* extract prevented the occurrence of methamphetamine toxic effects in SH-SY5Y cells after 24 hours. Moreover, the *S. angustifolium* extract decreased ROS levels and cytochrome C release and reduced BaX/Bcl2 ratio in cells treated by methamphetamine. On the whole, it seems that the S. *angustifolium* hydroalcoholic extract has the potential to increase cell survival through *in vitro* antioxidant and antiapoptotic activities.

## 1. Introduction

Signs of using herbal remedies can be found in ethnomedicine, current supplements, and medications. [1,2]. Due to the possibility of fraud in medicinal plants and variation of therapeutic agents concentration based on provider, season, and different variables, using concentrated herbal extracts is necessary. [3]. In 1893, methamphetamine (MA), n-methyl-1-phenylpropane-2-amine, was synthesized in Japan to treat idiopathic insomnia, attention deficit hyperactivity disorder, and narcolepsy [4]. Nowadays, MA abuse has become a major concern all over the world [5] because of its effects on central nervous system stimulation and causing euphoria, amplifying emotion, increment in alertness, alteration in self-esteem, and also increment sexuality [6]. It is shown that MA has a neurodegenerative effect on human brain and also can cause cardiomyopathy, myocardial problems, and respiratory failure [5]. MA-induced toxicity mechanisms involve several complex pathways; MA dramatically increases the production of reactive oxygen species (ROS) such as hydroxyl radicals (OH^−^), hydrogen peroxide (H_2_O_2_), and superoxide ions (O_2_^−^) by increasing the oxidation of dopamine [7]. In addition, numerous studies have shown that impaired mitochondrial metabolism plays a crucial role in MA-induced dopaminergic neurotoxicity by inhibiting the Krebs cycle and electron transfer chain and helping to induce oxidative stress. These effects lead to an imbalance between oxidants and antioxidants in nerve cells [8–11]. Moreover, gene expression analysis, *in-vitro* and *in-vivo* studies have revealed that MA can affect the apoptotic gene expression and cause cell death [5,12].

*Sargassum* is a genus of brown algae in the order *Fucales* that is widely distributed in the temperate and tropical oceans of the world [13,14]. It is claimed that different algae have had cytotoxic, antioxidant, antibacterial, antivirus, and antitoxin activities [15]. Some studies reported the antioxidant capacity of different species of *Sargassum* in an *in-vitro* model [16–18]. It is suggested that antioxidants compounds protecting nerve cells against MA-induced oxidative stress by reducing free radical production, maintaining glutathione (GSH) homeostasis, and inducing HO^−1^ expression [19]. Numerous studies have shown that the algae extract contains compounds such as amino acids, alkaloids, gallic acid, fatty acids, phenols, polysulfides, steroids, and aromatic compounds which have anticancer roles [20,21]. Namvar et al. reported that the S. *ilicifolium* methanolic extract could significantly affect human cancer cell lines while it does not have any toxicity effect on normal cells [22]. Also, Harada et al. reported selective cytotoxic activity of 47 species of algae in L1210 cells [23]. Moreover, anti-inflammatory effects of alginic acid [24], furans [25], and sargachromanol G [26] from S. *wightii*, S. *vulgare*, and S. *siliquastrum* have been revealed.

The aim of this study was to evaluate the effect of S. *angustifolium* hydroethanolic extract on MA-induced cytotoxicity in the SH-SY5Y dopaminergic cell line.

## 2. Methods

### 2.1. Preparation of the S. *angustifolium* Extract

The S. *angustifolium* algae were collected in December 2018 from the “Lengeh” port of Iran. They were identified by Dr. Askari, faculty member of the department of biology at the Shahid Bahonar University of Kerman. The samples were completely air-dried and pulverized by an electric mill. The dried S. *angustifolium* algae was soaked in 80% ethanol for 24 h and the extract was obtained by a maceration method. The extract solvent was omitted with a rotary evaporator at 40°C and material-dried in a 40°C oven. After dissolving the extract in dimethyl sulfoxide (DMSO), a solution with a concentration of 250 mg/ml was made. This solution was diluted in the sterile cell culture medium. The final hydroethanolic extract concentration was determined 3000 *μ*g/ml.

### 2.2. Cells and Cell Culture

The SH-SY5Y cell line was obtained from Pasteur Cell Bank of Iran. SH-SY5Y cells were cultured in Dulbecco's modified Eagle's medium (DMEM) medium containing glucose with 10% fetal bovine serum, penicillin (100 *μ*g/ml) and streptomycin (100 *μ*g/ml). The cells were incubated for 24 h at 37°C in a humidified 5% CO_2_.

### 2.3. MTT Assay

Living cells convert the MTT, a yellow dye, into formazan crystals, a purple dye, and determine the cellular metabolic activity [27]. SH-SY5Y cells were cultured in 96-well plates. SH-SY5Y cells were exposed to 20, 40, 80, 160, 320, 640 *µ*g/ml of *S. angustifolium* extract to assess the toxicity effect. To find the proper concentration of MA, the SH-SY5Y cells were treated with different concentrations of MA, and IC_50_ was determined. To evaluate the protective effect of the S. *angustifolium* extract on MA-induced toxicity, the cells were exposed simultaneously to 5 mM of MA and the different doses of the S. *angustifolium* hydroethanolic extract (20, 40, 80, 160, 320 and 640 *μ*g/ml). The light absorption of the purple dye was read by the ELISA at a wavelength of 490 nm.

### 2.4. Intracellular ROS Levels

The Human reactive oxygen species (ROS) ELISA Kit (Cat. No: MBS2515781) was used to measure ROS. This ELISA kit uses the Sandwich-ELISA principle. The micro-ELISA plate pre-coated with an antibody specific to Human ROS was used. Cells were seeded onto 96-well culture plates. 100 *μ*l of each sample (standard working solution, control group, cells treated with five mM·MA, cells treated with five mM·MA and doses of 80 and 160 *μ*g of the S. *angustifolium* extract) were added to plate wells and combined with 50 *μ*l of Biotin-Conjugate (primary antibody), then incubated for 60 min in 37°C. The supernatant was drained, and the wells were washed 3 times for 10 seconds. One hundred *μ*l of Streptavidin-HRP solution (secondary antibody conjugated to Horse radish peroxidase) was added to all wells and incubated at 37°C for 30 min. The wells were washed three times in 10 seconds. One hundred *μ*l of TMB substrate (tetramethylbenzidine, substrate-dye solution) was added to all wells and incubated for 15 minutes at 37°C in the dark. The absorbance was measured by ELISA-Reader at a wavelength of 450 nm.

### 2.5. Protein Extraction and Western Blot Analysis

To assess the ability of MA to induce apoptosis, BaX/Bcl2 ratio and cytochrome C were evaluated. The treated SH-SY5Y cells were lysed with TNE buffer (50 mM Tris-HCl pH 7.4, 150  mM NaCl, 1 mM ethylenediaminetetraacetic acid (EDTA), 1% Triton X-100, 1% sodium deoxycholate, 0.1% sodium dodecyl sulfate (SDS), and 1 mM (PMSE) followed by centrifugation at 13,000 rpm for 4 min at 10°C. The supernatant was collected. Equal amounts of protein (20 *μ*l at a concentration of 40 *μ*g) were separated by 12% SDS-polyacrylamide gel electrophoresis and transferred onto polyvinylidene difluoride membranes. The membranes were blocked with nonfat milk powder (with a concentration of 2% in TBST solution) and washed three times with Tris-buffered saline containing 0.1% TBST. After washing, the membranes were incubated at 4°C overnight with the following appropriate antibodies: Cytochrome c (1 :  1,000), Bax (1 : 1,000), and Bcl-2 (1 :  1,000) and washed three times with TBST, and incubated secondary antibody: goat anti-mouse (1 :  15,000) and washed 3 times with TBST. The membranes were visualized via enhanced chemiluminescence (ECL Advance Kit).

### 2.6. Statistical Analysis

Data are expressed as the means ± standard error of the mean (SEM) and were analyzed using SPSS Statistics 23.0 software. Groups were compared using the Student-Newman–Keul's and one-way analysis of variance (ANOVA). The significance level was set at *p* value < 0.05. Figures were designed using Excel 2019 software.

## 3. Result

### 3.1. Effect of Different Concentrations of  MA on SH-SY5Y Cell Survival

**Figure 1:**
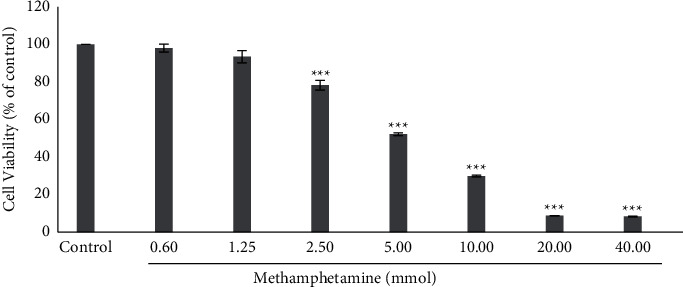
Effect of different concentrations of MA on SH-SY5Y cell survival.

As shown in Figure 1, the MA lethal effect was dependent on its concentration. Since MA showed a significant toxic effect at a concentration of 5 mM and approximately killed 50% of the cells, this concentration was considered IC_50_ and was used in other stages of the experiment.

Results are expressed as the means ± standard error of the mean (SEM). ^*∗∗∗*^, *P* < 0.001 compared with the control (untreated) group.

### 3.2. Effect of Different Concentrations of the S. *angustifolium* Extract on SH-SY5Y Cell Survival

**Figure 2:**
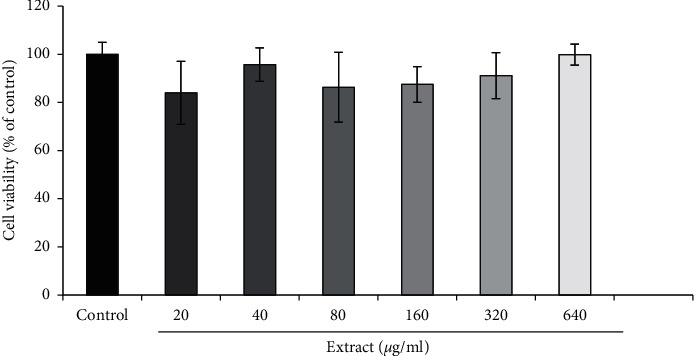
Effect of different concentrations of the *S. angustifolium* hydroethanolic extract on SH-SY5Y cell survival.

According to the results of MTT test, the S. *angustifolium* extract did not show toxicity in any dose (Figure 2).

Results are expressed as the means ± standard error of the mean (SEM). No significant difference was seen between the groups treated with the S. *angustifolium* extract and the control (untreated) group.

### 3.3. Effect of Different Concentrations of the S. *angustifolium* Extract on SH-SY5Y Cell Survival in the Presence of MA

**Figure 3:**
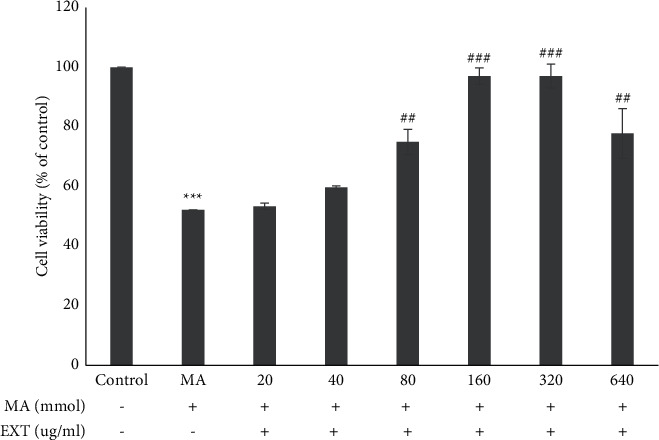
Effect of different concentrations of the *S. angustifolium* extract on SH-SY5Y cell survival in the presence of MA.

The results of MTT test showed that the S. *angustifolium* extract in doses 80, 160, 320, and 640 *μ*g/ml prevented occurrence of MA toxic effects after 24 hours (Figure 3).

Results are expressed as the means ± standard error of the mean (SEM). ^*∗∗∗*^, *P* < 0.001 compared with the control (untreated) group. ^###^*P* < 0.001 and ^##^*P* < 0.01 compared with the MA group.

### 3.4.  The  Effect of the S. *angustifolium* Extract on ROS Produced by  MA  in SH-SY5Y Cells

**Figure 4:**
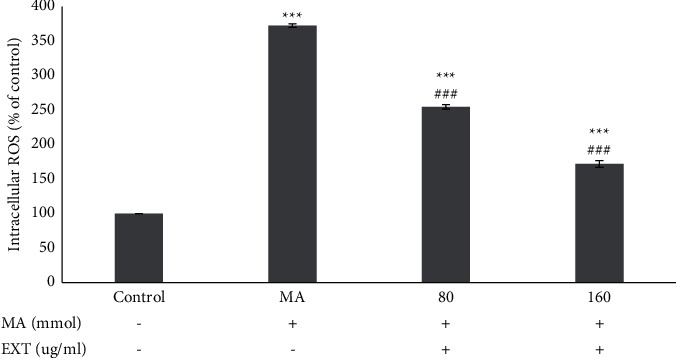
Effect of the S. *angustifolium* extract on ROS produced by MA in SH-SY5Y cells.

According to the results (Figure 4), ROS levels in the MA-treated group significantly increased in comparison to the control group (*P* < 0.001). The results of ROS assay in MA-treated cells with doses of 80 and 160 *μ*g/ml S. *angustifolium* extracts were significantly different from the MA group (*P* < 0.001) and the S. *angustifolium* extract could prevent the production of intracellular ROS.

Results are expressed as the means ± standard error of the mean (SEM). ^*∗∗∗*^, *P* < 0.001 compared with the control (untreated) group. ^###^*P* < 0.001 compared with the MA group.

### 3.5. Protein Extraction and Western Blot Analysis

**Figure 5:**
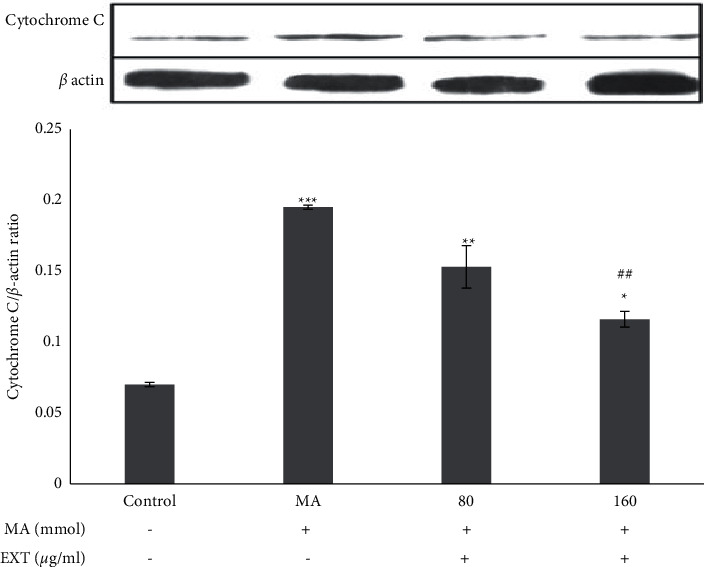
Effect of the S. *angustifolium* extract on cytochrome C protein expression in SH-SY5Y cells.

**Figure 6:**
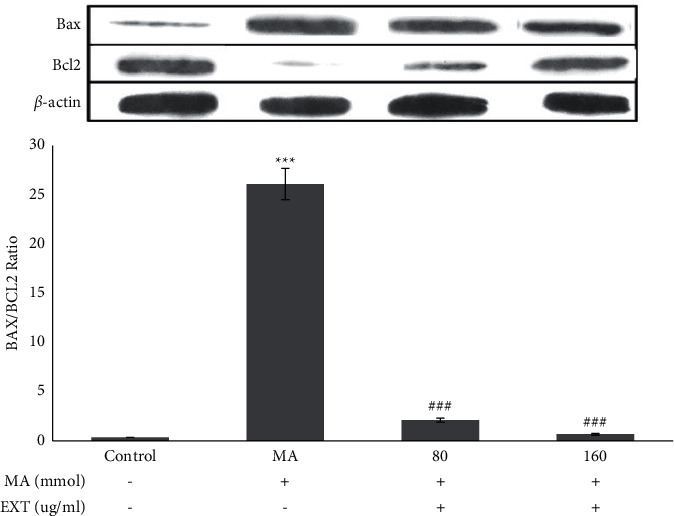
Effect of the S. *angustifolium* extract on Bax/Bcl-2 protein expression in SH-SY5Y cells.

The release of cytochrome C in SH-SY5Y cells exposed to MA was significantly different from the control group (treated with normal medium) (*P* < 0.001). As shown in Figure 5, when cells were treated simultaneously with 5 mM·MA and different doses of the *S. angustifolium* extract (80 and 160 *μ*g/ml), the amount of cytochrome C was significantly reduced by the MA group. In SH-SY5Y cells treated with MA, the ratio of Bax/Bcl2 increased significantly compared to cells cultured in a normal medium (*P* < 0.001). When MA-treated cells received 80 and 160 *μ*g/ml of the S. *angustifolium* extract, the Bax/Bcl2 ratio was significantly reduced compared to MA-treated cells (*P* < 0.001) (Figure 6).

*β*-actin served as a protein loading control. The relative level of cytochrome C was normalized with *β*-actin. Results are expressed as the means ± standard error of the mean (SEM). ^*∗*^, *P* < 0.05, ^*∗∗*^, *P* < 0.01 and ^*∗∗∗*^, *P* < 0.001 compared with the control (untreated) group. ^##^*P* < 0.01 compared with the MA group.

The relative levels of Bax and Bcl2 were normalized with *β*-actin. Results are expressed as the means ± standard error of the mean (SEM). ^*∗∗∗*^, *P* < 0.001 compared with the control (untreated) group. ^###^*P* < 0.001 compared with the MA group.

## 4. Discussion

Mitochondrial metabolism disturbance plays a vital role in MA-induced dopaminergic neurotoxicity by inhibiting the Krebs cycle and electron transport chain and contributing to oxidative stress [28]. Due to lipophilic and cationic structure, MA could easily enter the mitochondria. Superoxide ion and hydrogen peroxidase formation have been identified as the main cause of MA-induced neurotoxicity [29,30]. This phenomenon is clinically important because it could ultimately lead to destructive neurological effects in the brain due to mitochondrial dysfunction, induction of intracellular oxidants, caspase activation, and apoptotic neuronal death [10]. In the present study, MTT assay was used to evaluate the protective effects of the S. *angustifolium* hydroethanolic extract on cytotoxicity induced by MA. Results demonstrated that the S. *angustifolium* extract in doses of 80, 160, 320, and 640 could prevent the occurrence of MA toxic effects.

MA leads to the formation of intracellular ROS by altering intracellular calcium signaling and weakening antioxidant defenses [31]. Following the use of antioxidants, increasing the expression of Cu-Zn-SOD and MnSOD (superoxidases) and decreasing the amount of ROS, MA-induced toxicity is attenuated [32]. In the present study, the results of ROS assay in cells treated simultaneously with MA and 80 and 160 *μ*g/ml of S. *angustifolium* extract showed that the S. *angustifolium* extract significantly inhibited intracellular ROS production. The effect of different species of *Sargassum* on reducing intracellular ROS production has been reported previously [24,33]. According to studies, *Sargassum* contains terpenoids that have biological activities such as cytotoxicity in cancer cells and antioxidant activity [15,34]. In addition, the brown algae of the genus *Sargassum* have unique secondary metabolites such as plastoquinone [35], chromanols [36], polysaccharides [37], and fecosterol [38], which have been introduced as a good option for protection against free radicals. A study by Babakhani et al. reported that the phenolic compounds of S. *angustifolium* include gallic acid, protocatechuic acid, gentisic acid, and hydroxybenzoic acid which makes it seem that this alga is a rich source of antioxidant compounds [39]. Numerous studies have shown that fucoidan, stypoldione, terpenes, sterols, fatty acids, and phenolic compounds have great potential for anticancer and cytotoxic activities [40,41]. Due to the fact that the S. *angustifolium* extract contains large amounts of phenolic compounds, which is among the most important antioxidants [42], it is possible that the protective effect and cell viability of S. *angustifolium* hydroethanolic extract be related to its phenolic compounds.

MA-induced neurodegeneration associated with mitochondrial apoptosis [43]. The presence of reactive oxygen and nitrogen species indicates an increase in the expression of apoptotic promoting proteins: Bax, Bad, and Bid as well as a decrease in expression of antiapoptotic proteins: Bcl-2 and Bcl-XL [44], and increased Bax/Bcl-2 ratio indicates release of cytochrome C from mitochondria [45]. Subsequently, cytochrome C binds with apoptotic protease-activating factor 1 (Apaf-1), and in the execution phase of apoptosis, activates the initiator caspase [6,46,47]. According to the abovementioned facts, oxidative stress caused by MA is one of the main factors in inducing apoptosis. Inhibition of these factors plays an important role in preventing the progression of apoptosis.

The present study results showed that the *S. angustifolium* extract has a significant effect on reducing the Bcl2/Bax ratio and reducing the release of cytochrome C in SH-SY5Y cells exposed simultaneously to 5 mM·MA and 80 and 160 *μ*g/ml of S. *angustifolium* extract. Therefore, decreased expression of apoptotic proteins could be considered as one of the possible mechanisms of the S. *angustifolium* extract in reducing apoptosis. In addition, the anti-inflammatory activity of *Sargassum* species extract has been reported through inhibition of COX-2, TNF-*α*, IL-6, PGE2, and inhibition of NF-*κ*B nuclear translocation [24,38]. Anti-inflammatory compounds, via inhibition of the expression of inflammatory mediators and apoptosis proteins, play a role in controlling their function [48]. So, according to the abovementioned statements, one of the possible mechanisms of S. *angustifolium* extract in reducing the expression of apoptotic proteins could probably be the inhibition of inflammatory factors.

## 5. Conclusion

The S. *angustifolium* hydroethanolic extract decreased MA-induced toxicity in the SH-SY5Y cells line by reducing intracellular ROS, cytochrome C, and Bax to Bcl2 ratio, and significantly increased SH-SY5Y cells survival in the current *in vitro* study. Further studies are needed to identify the most effective and potent compounds in the S. *angustifolium* extract which play a key role in cellular protection.

## Data Availability

The data used to support the findings of this study are available from the corresponding author on request.
